# Association between swallowing dynamics, tongue pressure and pulmonary function in patients with idiopathic pulmonary fibrosis

**DOI:** 10.1186/s12890-024-03117-3

**Published:** 2024-07-04

**Authors:** Paula Vasconcellos, Thiago Thomaz Mafort, Marcelo Ribeiro-Alves, Cláudia Henrique da Costa

**Affiliations:** 1 Pedro Ernesto University Hospital, Boulevard 28 de Setembro, 77 – Vila Isabel, Rio De Janeiro, Brazil; 2 Pulmonology Discipline, Pedro Ernesto University Hospital, Boulevard 28 de Setembro, 77 – Vila Isabel, Rio de Janeiro, Brazil; 3 Laboratório de Pesquisa Clínica em DST-AIDS, Instituto Nacional de Infectologia Evandro Chagas, Oswaldo Cruz – FIOCRUZ - Rua Leopoldo Bulhões, Manguinhos, Rio de Janeiro, 1480 Brazil

**Keywords:** Swallowing, Idiopathic pulmonary fibrosis, Tongue pressure, Oropharyngeal swallow

## Abstract

**Background:**

Swallowing is a complex process that requires the coordination of muscles in the mouth, pharynx, larynx, and esophagus. Dysphagia occurs when a person has difficulty swallowing. In the case of subjects with respiratory diseases, the presence of oropharyngeal dysphagia potentially increases lung disease exacerbations, which can lead to a rapid decline in lung function. This study aimed to analyze the swallowing of patients with idiopathic pulmonary fibrosis (IPF).

**Methods:**

Patients with IPF were evaluated using the Eating Assessment Tool (EAT-10), tongue pressure, the Timed Water Swallow Test (TWST), and the Test of Mastication and Swallowing Solids (TOMASS). The findings were related to dyspnea severity assessed by the modified Medical Research Counsil (mMRC) score; the nutritional status screened with Mini Nutritional Assessment (MNA) tool; and pulmonary function tests, specifically spirometry and measurement of the diffusing capacity for carbon monoxide (DLCO), the maximal inspiratory pressure (PImax), and the maximal expiratory pressure (PEmax).

**Results:**

The sample consisted of 34 individuals with IPF. Those who exhibited swallowing modifications scored lower on the MNA than those who did not (9.6 ± 0.76 vs. 11.64 ± 0.41 points; mean difference 1.98 ± 0.81 points; *p* = 0.02). They also showed poorer lung function when considering the predicted force vital capacity (FVC; 81.5% ± 4.61% vs. 61.87% ± 8.48%; mean difference 19.63% ± 9.02%; *p* = 0.03). The speed of liquid swallowing was altered in 31of 34 of the evaluated subjects (91.1%). The number of liquid swallows correlated significantly with the forced expiratory volume in 1 s (FEV1)/FVC ratio (*r* = 0.3; *p* = 0.02). Solid eating and swallowing assessed with the TOMASS score correlated with lung function. The number of chewing cycles correlated negatively with PImax% predicted (*r* = -0.4; *p* = 0.0008) and PEmax% predicted (*r* = -0.3; *p* = 0.02). FVC% predicted correlated with increased solid swallowing time (*r* = -0.3; *p* = 0.02; power = 0.6). Swallowing solids was also impacted by dyspnea.

**Conclusion:**

Patients with mild-to-moderate IPF can present feeding adaptations, which can be related to the nutritional status, lung function, and the severity of dyspnea.

## Introduction

Swallowing is a complex process that requires the coordination of over 30 pairs of muscles in the mouth, pharynx, larynx, and esophagus [1], with the goal of safely and efficiently transporting the food bolus from the oral cavity to the stomach. The pharynx serves two important and distinct functions: respiration and swallowing. The passage of food from the oral cavity to the pharynx without invading the lower airway is one of the major challenges of swallowing. To ensure safe swallowing, coordination between respiration and swallowing is necessary [2]. Difficulty and/or alteration during the swallowing process is referred to as dysphagia [3, 4], which can be caused by structural or functional alterations. In individuals with chronic obstructive pulmonary disease (COPD), the presence of oropharyngeal dysphagia may increase disease exacerbations [5]. Individuals with COPD are more likely to experience a rapid decline in lung function, leading to a higher number of hospitalizations [1].

Interstitial lung diseases are characterized by involvement of the lung interstitium. There is activation of fibroblasts located in the interalveolar space and an increase in collagen production, rendering the lungs increasingly less compliant and with progressive impairment in their gas exchange [6]. Among idiopathic interstitial lung diseases, interstitial pulmonary fibrosis (IPF) is the most frequently diagnosed. Currently, the adjusted global incidence of IPF ranges from 0.09 to 1.30 per 10,000 people, and the prevalence ranges from 0.33 to 4.51 per 10,000 people [7]. Despite being a relatively rare disease, IPF is of great clinical importance due to its severity [8]. The natural history of the disease can vary, but the survival without treatment is of 2–5 years [8]. Between 20% and 40% of individuals survive for 5 years or more [9]. Factors that may negatively impact the nutritional status in the progression of IPF include increased respiratory muscle burden, the release of inflammatory mediators, the presence of hypoxemia, and physical inactivity [10]. Malnutrition is common among individuals with IPF and is associated with increased morbidity and mortality [11]. The nutritional status has been shown to be a predictor of poor outcomes in some lung diseases such as COPD and tuberculosis, but its clinical impact in individuals with IPF is not yet fully understood [10, 12].

Given the close relationship between respiration and swallowing, due to the shared anatomical structures involved in both processes and the need for synchrony between them to ensure safe and efficient function, studies have evaluated swallowing function in individuals with chronic respiratory diseases [13, 14]. However, there is a knowledge gap regarding the impact of respiratory, nutritional, and muscle mass alterations on swallowing in individuals with IPF.

The aim of this study was to analyze the swallowing of patients with IPF. We also evaluated swallowing efficiency, mastication, and clinical signs of airway invasion and assessed the association between swallowing dynamics, participant anthropometrics, and lung function. As a secondary aim, we assessed the nutritional status of the patients.

## Methods

Individuals diagnosed with IPF according to the guidelines set by the American Thoracic Society (ATS), the European Respiratory Society (ERS), the Japanese Respiratory Society (JRS), and the Latin American Thoracic Association (ALAT) [15] were invited to participate. They were enrolled from the interstitial diseases outpatient clinic at the Piquet Carneiro Polyclinic, State University of Rio de Janeiro (UERJ). Individuals with a history of head and neck cancer, dementia, neuromuscular diseases, or stroke were excluded, as well as those who were experiencing a pulmonary infection or exacerbation of IPF during the assessment period, or those unable to undergo the planned procedures.

The following investigations were performed:

1. Initial interview. The individuals were asked if they had experienced any swallowing difficulties and if they had made any adaptations during feeding (e.g., moistening the diet, pauses during meals, control of the liquid intake rate and volume from the cup, and intake of liquids during solid food intake).
2. Questionnaires. The Modified Medical Research Council (mMRC) [16], the Mini Nutritional Assessment (MNA) [17], and the Eating Assessment Tool (EAT-10) [18] were applied. The mMRC [16] is a validated questionnaire that grades dyspnea. It uses five questions about the presence of dyspnea during daily activities and is scored from 0 to 4, with higher values indicating more severe dyspnea. Any individuals with a score of ≥ 2 is considered to be symptomatic. Nutritional screening was performed using the MNA [17], a tool recommended by the European Society of Parenteral and Enteral Nutrition, the International Association of Gerontology, and the International Academy of Nutrition and Aging [19]. Based on the questionnaire score, individuals were categorized as malnourished (0–7 points), at risk of malnutrition (8–11 points), or as having a normal nutritional status (12–14 points), as established in the questionnaire validation [17]. The EAT-10 is a widely used questionnaire for dysphagia assessment [18]. It consists of 10 statements, and individuals rate themselves on a scale of 0–4 points for each statement. The total score ranges from 0 to 40, with a score of ≥ 3 suggesting risk of dysphagia [18].
3. Tongue pressure measurement. A speech-language pathologist specialized in dysphagia measured tongue pressure by using the Biofeedback Pro-Fono: Lip and Tongue Pressure device (Pro Fono, Carapicuiba, SP, Brazil). The equipment consists of an air bulb connected to a pressure sensor through a flexible plastic tube. During the examination, the individuals remained seated in a comfortable chair with their feet on the ground and their heads parallel to the horizontal plane. They were instructed to position the bulb on their tongue and exert pressure with their tongue against the palate for 2–5 s, on three occasions spaced at 30-second intervals, as recommended by the manufacturer. After three measurements had been recorded, the device’s software generated a graph with the average force obtained on each occasion and the overall average of the three. The measured tongue pressure is expressed in kilopascals (kPa). To date, there are no reference values ​​for tongue pressure measured using this device, so individuals were stratified as above or below the median.
4. Swallowing assessment. The Timed Water Swallow Test (TWST) [20] and the Test of Mastication and Swallowing Solids (TOMASS) [21] were used for swallowing assessment. Both assessments were performed with the individuals seated, after instructing them to swallow water and a biscuit as they would normally do. The TWST [20] was performed with 150 mL of water, and the number of swallows was counted by observing the movement of the thyroid cartilage. The total ingestion time was measured, and several ratios—volume/time (TWST v/t), volume/swallow (TWST v/s), and time/swallow (TWST t/s)—were calculated. Any alterations such as coughing, choking, throat clearing, the sensation of food being stuck, or a wet voice were also recorded. The results of the TWST [20] were analyzed, based on the normal values according to sex and age range for the volume/time, volume/swallow, and time/swallow ratios proposed by Sarve et al. [22]. The test was considered abnormal if at least one of the ratios was outside the normal range. The TOMASS [21] is designed to provide the examiner with objective data on the efficiency of oral phase function and solid bolus ingestion. The assessment involved offering a cream cracker biscuit measuring 5.5 cm × 5.5 cm and weighing 5 g. The total ingestion time, number of bites, number of swallows per bite (TOMASS s/b), and number of masticatory cycles (TOMASS mc) necessary to ingest the biscuit were recorded. Any alterations such as coughing, choking, throat clearing, a sensation of food being stuck, or a wet voice were also recorded. The reference data available for the TOMASS [21] are related to the size and weight of the biscuit used, but biscuits with established reference data are not available in Brazil. Therefore, the median total time was calculated, and the individuals were stratified as above or below this median.
5. Pulmonary function assessment. Spirometry and measurement of the diffusing capacity for carbon monoxide (DLCO), the maximal inspiratory pressure (PImax), and the maximal expiratory pressure (PEmax) were used to assess pulmonary function. These tests were conducted using an HDpft 3000 device (nSpire Health Inc., Longmont, CO, USA). The following measures were calculated: the percentage of the predicted forced vital capacity (FVC% predicted), the percentage of the predicted forced expiratory volume in 1 s (FEV1% predicted), and the FEV1/FVC ratio. The theoretically predicted spirometry values ​​were described by Knudson et al. [23]. DLCO, PImax, and PEmax were measured by following the standardization and interpretation of the ATS [24], and the Neder equations were adopted [25, 26].

All assessments were conducted on the same day. The pulmonary function assessment was performed by a pulmonologist, and the others were performed by a speech-language pathologist specializing in dysphagia.

### Statistical analysis

The sociodemographic and clinical characteristics of the sample are described with either the mean ± standard deviation (SD) for continuous numerical variables, or with the absolute (relative) frequency for nominal variables. Multiple linear fixed-effects models assessed the mean marginal differences between/among levels for the nominal variables. Confounding variables (i.e., age, sex, body mass index [BMI], and the use of specific medications) were included as covariates. The graphics present the estimated mean marginal effects and their 95% confidence intervals. The Tukey honest significant difference (HSD) method was used to correct p-values ​​by the number of comparisons. Statistical power for the mean differences was estimated by calculating Cohen’s d [27]—that is, the mean differences divided by the pooled standard deviation, assuming unpaired two-tailed t-tests with a significance level (probability of a type I error) of 0.05. Pearson’s adjusted linear correlation analysis, including confounding variables, was used to determine the correlations between the continuous numerical variables. The statistical power for the correlations was estimated by calculating the Z′ transformation of the correlation coefficients [27] —that is, Z′ = arctanh(r)—and a bias correction. Some continuous numerical variables were log-transformed for normalization. Statistical significance was set at the level of 5%. R version 4.2.1 and the packages lme4, emmeans, and pwr were used to perform the statistical analyses.

## Results

**Fig. 1:**
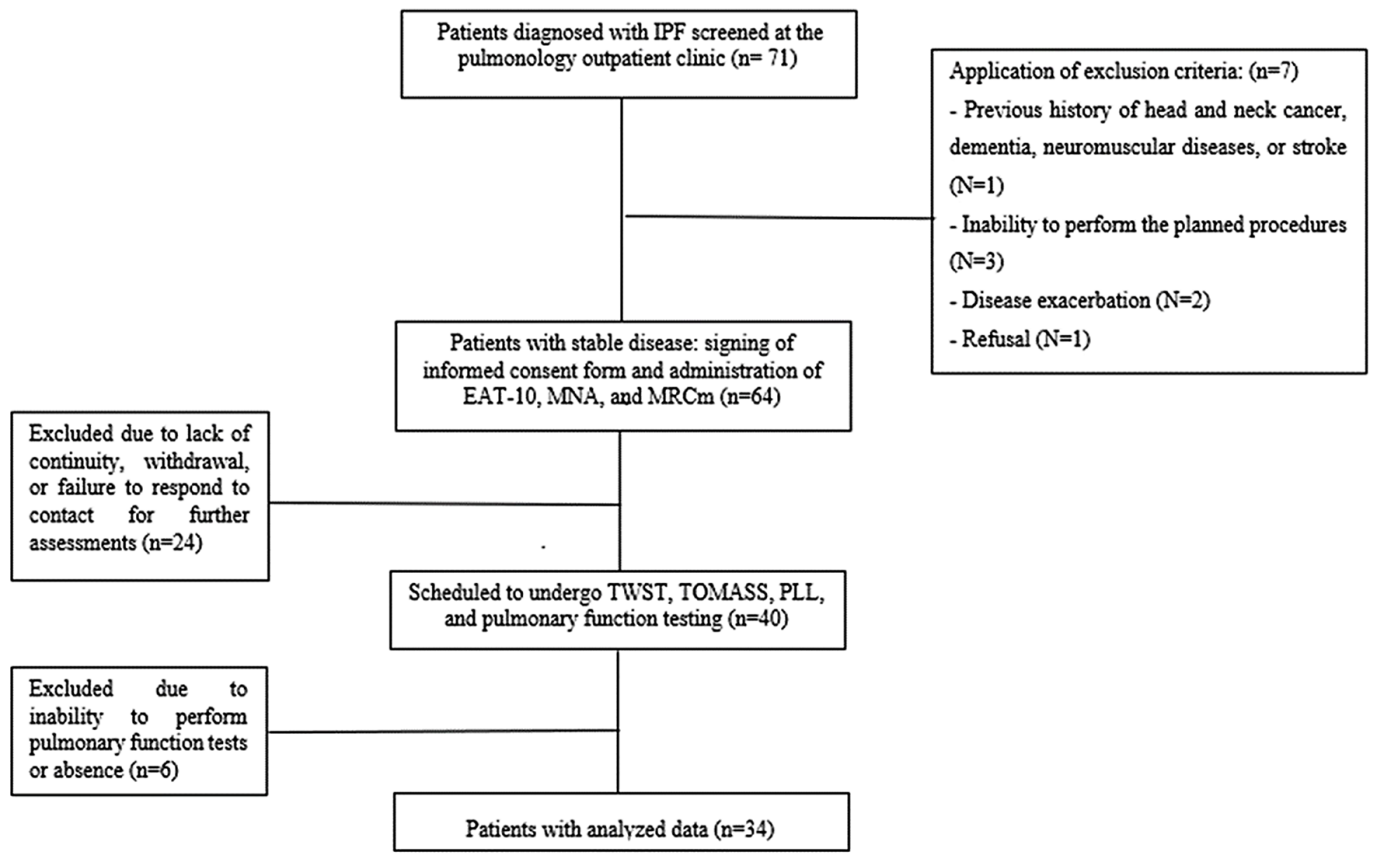
Methodological strategy. The pulmonary function tests including spirometry and measurement of the diffusing capacity for carbon monoxide (DLCO), the maximal inspiratory pressure (PImax), and the maximal expiratory pressure (PEmax). *Abbreviations* EAT-10 = Eating Assessment Tool; IPF = idiopathic pulmonary fibrosis; mMRC = Medical Research Council; MNA = Mini Nutritional Assessment; PLL = tongue pressure measurement; TOMASS = Test of Mastication and Swallowing Solids; TWST = Timed Water Swallow Test

We screened 71 individuals diagnosed with IPF and receiving care at the pulmonology clinic for the study. We excluded 37 individuals, leaving a final sample size of 34 individuals as outlined in Fig. 1. The analyzed sample consisted of 27 (79.4%) male individuals and 7 (20.6%) female individuals, with a mean ± SD age of 75 ± 6.6 years.

Of the 34 individuals included in the analysis, 18 (52.9%) presented with dyspnea (mMRC ≥ 2), and 6 (17.6%) of them required supplemental oxygen. 50% of the sample had a normal FVC% predicted. Six individuals (17.6%) had mild-to-moderate disease severity according to the classification by Kolb and Collard [28]. After applying the MNA, 18 individuals (52.9%) had a normal nutritional status, which means that they scored *≥* 12 points. The mean BMI was 23.9 ± 3.95 kg/m², with 52.9% of subjects classified as eutrophic. The spirometric data, patient characteristics, and test results are described in Table 1.

**Table 1:** The spirometric data, patient characteristics, and test results

Characteristic	(*n* = 34)
**Age in years, mean** ± **SD**	75 ± 6.6
**Sex**	
Male, n (%) Female, n (%)	27 (79%) 7 (20%)
**mMRC**	
0 or 1, n (%) ≥ 2, n (%)	16 (47%) 18 (53%)
**MNA**	
Normal nutritional status, n (%) Nutritional risk, n (%) Malnutrition, n (%)	18 (53%) 15 (44%) 1 (3%)
**BMI, mean** ± **SD (kg/m²)** ≥ 18.5 but ≤ 25, n (%) ≥ 25 but < 30, n (%) ≥ 30, n (%)	23.9 ± 3.95 18 (53%) 13 (38%) 3 (9%)
**FVC**, mean ± SD (% of predicted)	79.5 ± 22.68
**FEV1**, mean ± SD (% of predicted)	85 ± 26.61
**FEV1/FVC**, mean ± SD (%)	85 ± 26.61
**DLCO**, mean ± SD (% of predicted)	52 ± 21.4
**PImax**, mean ± SD (% of predicted)	73.5 ± 30.81
**PEmax**, mean ± SD (% of predicted)	50 ± 19.9
**EAT-10 score**	
< 3, n (%) ≥ 3, n (%)	29 (85.3%) 5 (14.7%)
**TWST volume per swallow**	
Normal, n (%) Abnormal, n (%)	5 (14.7%) 29 (85.3%)
**TWST time per swallow**	
Normal Abnormal	10 (29.4%) 24 (70.6%)
**TWST volume/time**	
Normal, n (%) Abnormal, n (%)	3 (8.8%) 31(91.2%)
**TOMASS – total time**, mean ± SD (seconds)	54.5 ± 27.7
**TOMASS – number of masticatory cycles**, mean ± SD	26 ± 17.32
**TOMASS – number of bites**, mean ± SD	3 ± 1.23
**TOMASS – swallows per bite**, mean ± SD	1 ± 0.69
**Tongue pressure**, mean ± SD (kPa)	55.04 ± 16.11

Legend: mMRC = Modified Medical Research Council; MNA = Mini Nutritional Assessment; BMI = body mass index; FVC = forced vital capacity; FEV1 = forced expiratory volume in 1 s; DLCO = diffusing capacity for carbon monoxide; PImax = maximum inspiratory pressure; PEmax = maximum expiratory pressure; EAT-10 = Eating Assessment Tool; TWST = Timed Water Swallow Test; TOMASS = Test of Mastication and Swallowing Solids; SD = standard deviation

**Fig. 2:**
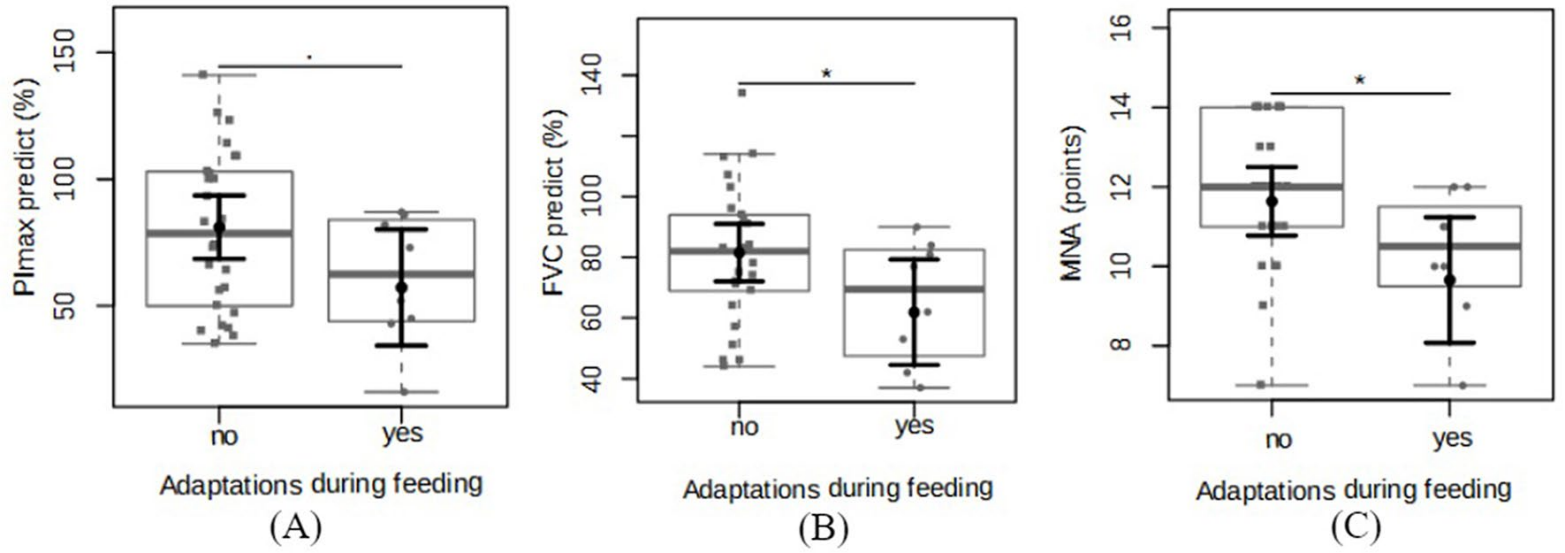
Differences in the (**A**) predicted maximum inspiratory pressure (PImax), (**B**) the predicted forced vital capacity (FVC), and (**C**) the Mini Nutritional Assessment (MNA) between patients with interstitial pulmonary fibrosis (IPF) and with or without feeding adaptations. The sample distributions are plotted in the box plots and strip charts in gray. In black, the central circle represents the estimated mean marginal effect expected for each group estimated from linear fixed-effects models. The confounding variables were age, sex, body mass index, and the use of specific medications. The black horizontal bars represent the 95% confidence intervals of the expected mean marginal effects per group

We observed feeding adaptations during the swallowing assessment in 8 of the 34 individuals (23.5%). These included diet moistening (2.9%), pauses during meals (5.9%), control of the liquid intake rate and volume from the cup (5.9%), and intake of liquids during solid food intake (8.8%). The mean muscle strength assessed by the predicted PImax was significantly higher in individuals who did not make adaptations compared with the individuals who made adaptations (81.03% ± 6.09% vs. 57.21% ± 11.20%; mean difference 23.82 ± 11.92; *p* = 0.05; power = 0.4) (Fig. 2A). The individuals who made feeding adaptations showed poorer lung function when considering FVC% predicted (81.5% ± 4.61% vs. 61.87% ± 8.48%). The mean difference was 19.63% ± 9.02% (*p* = 0.03; power = 0.5) (Fig. 2B). The individuals who made swallowing modifications scored lower on the MNA (9.6 ± 0.76 points) than those who did not make these adaptations (11.64 ± 0.41 points). The mean difference was 1.98 ± 0.81 points (*p* = 0.02; power = 0.6) (Fig. 2C).

**Fig. 3:**
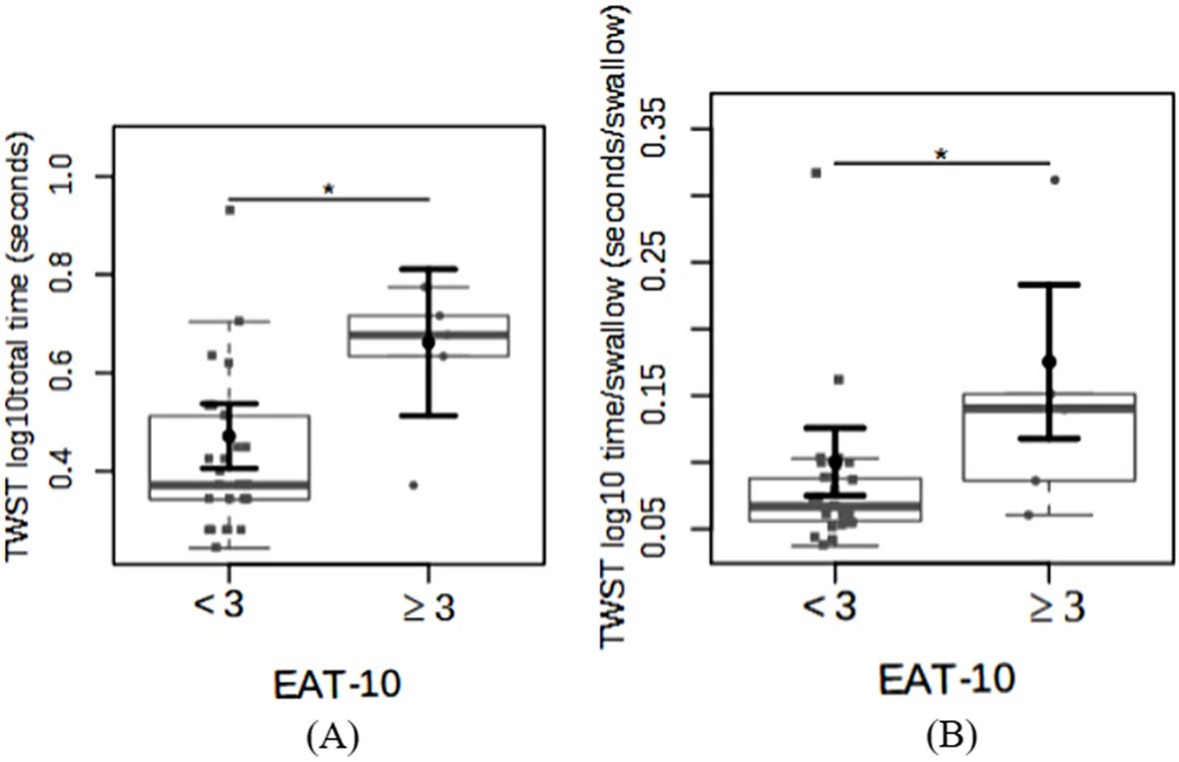
Differences (log10 fold-changes) for (**A**) the Timed Water Swallow Test (TWST) total time (log10) and (**B**) TWST total time (log10) per swallow based on the individuals with an Eating Assessment Tool (EAT-10) score of liquid swallowing either below 3 (lower risk of dysphagia) or above 3 (higher risk of dysphagia). The data are plotted in box plots and strip charts in gray. In black, the central circle represents the estimated mean marginal effect expected for each group estimated from linear fixed-effects models. The confounding variables were age, sex, body mass index, and use of specific medications. The black horizontal bars represent the 95% confidence intervals of the expected mean marginal effects per group

Analysis of the EAT-10 scores indicated a risk of dysphagia in 5 of the 34 individuals (14.7%). The average score of these 5 individuals was 3.8, very close to the normal value. However, all individuals with an EAT-10 score ≥ 3 showed alterations in liquid swallowing, assessed through the TWST (Fig. 3A, B). The mean total liquid swallowing time (log_10_) in individuals without a risk of dysphagia was 0.4 ± 0.03 s, while in those at risk, it was significantly higher at 0.66 ± 0.07 s (log_10_ fold-change 0.19 ± 0.07; *p* = 0.01; power = 0.7). Individuals at risk of dysphagia also exhibited a significantly longer time (log_10_) on each swallow compared with individuals not at risk of dysphagia (0.17 ± 0.02 vs. 0.10 ± 0.01; log_10_ fold-change 0.07 ± 0.02; *p* = 0.01; power = 0.7).

**Fig. 4:**
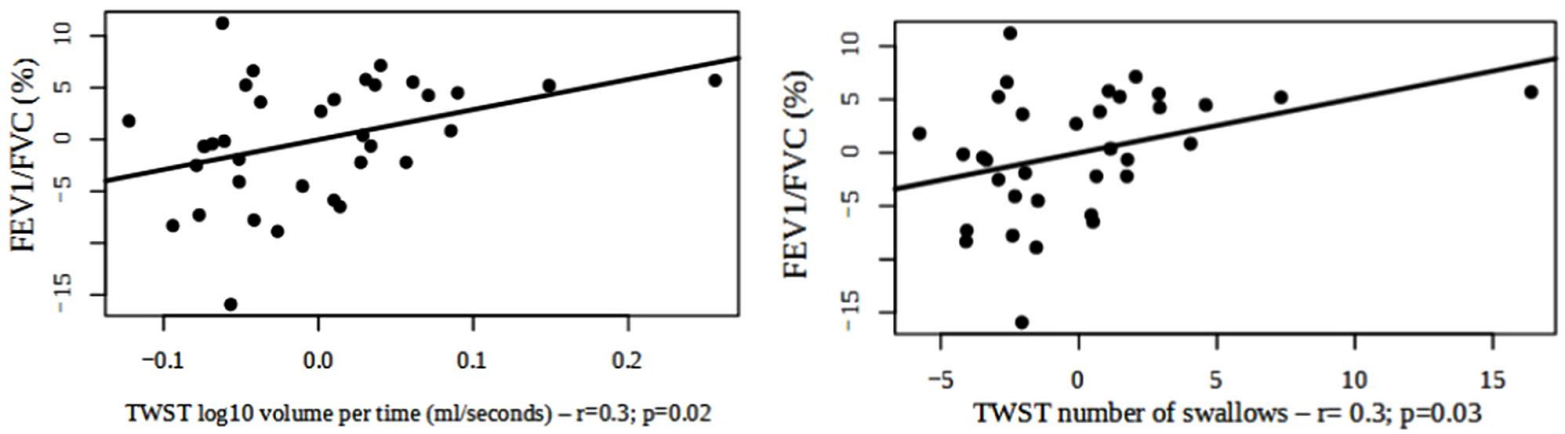
Correlation between the ratio of the forced expiratory volume in 1 s to the forced vital capacity (FEV1/FVC) and liquid swallowing. The confounding variables were age, sex, body mass index, and the use of specific medications. *Abbreviations* TWST = timed water swallow test

Liquid swallowing (TWST) was altered in all individuals in the sample; all three ratios calculated based on this method were altered in 47% of the individuals. The speed of liquid swallowing was altered in 31 of the 34 evaluated individuals (91.1%). Liquid swallowing correlated with lung volume (FVC). However, liquid swallowing correlated inversely and significantly with the FEV1/FVC ratio (*r* = 0.3; *p* < 0.05; power = 0.6 and 0.5, respectively), supporting the hypothesis that there is a relationship between functional severity and difficulty in liquid swallowing (Fig. 4).

**Fig. 5:**
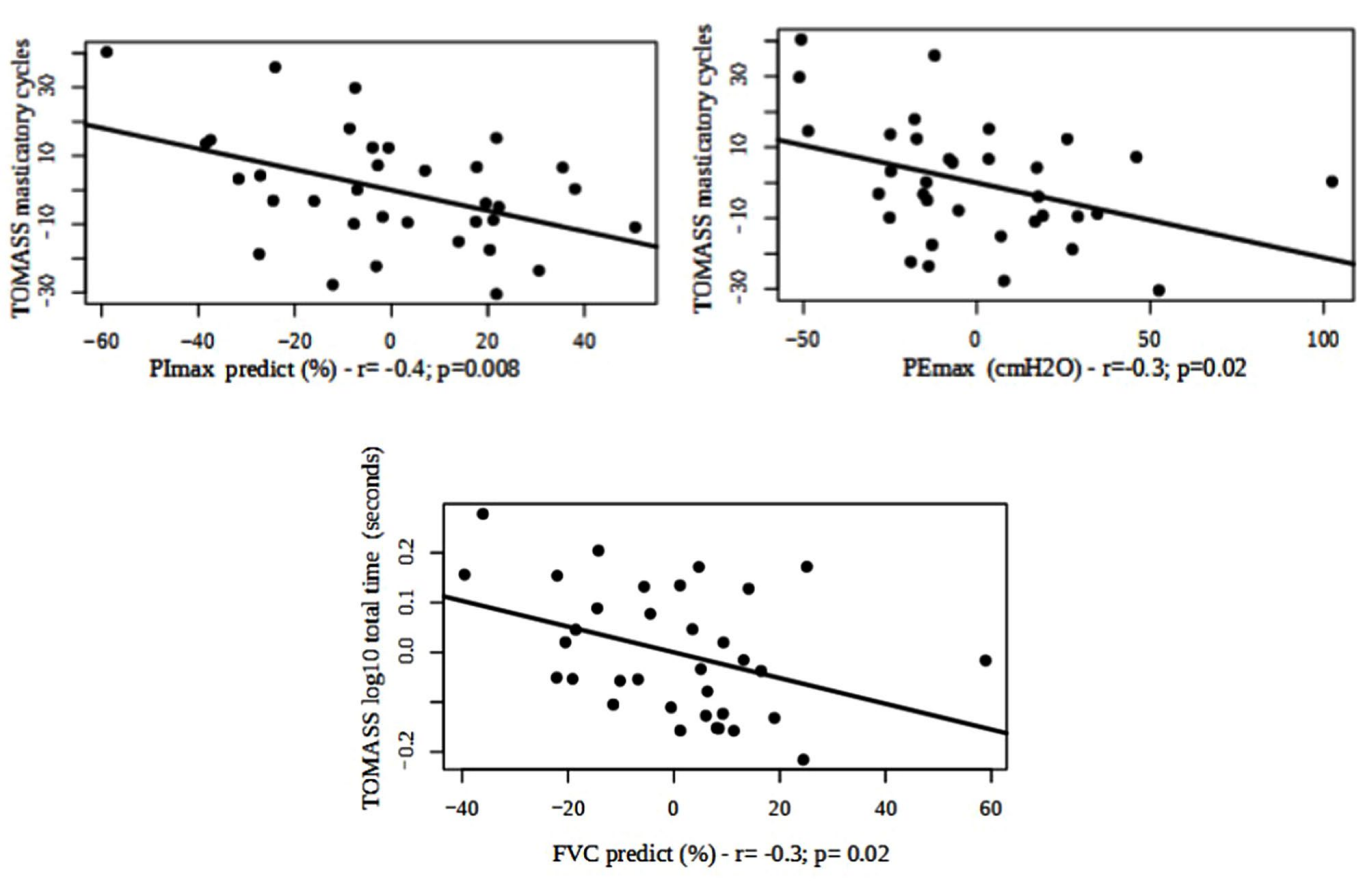
Correlation between pulmonary function and solid swallowing. The confounding variables were age, sex, body mass index (BMI), and the use of specific medications. *Abbreviations* FVC = forced vital capacity; PImax predict = predicted maximum inspiratory pressure; PEmax = maximum expiratory pressure; TOMASS = Test of Mastication and Swallowing Solids

We used the TOMASS score to analyze solid eating and swallowing. When correlating the total time and the number of chewing cycles, we observed a correlation with pulmonary function when considering FVC% predicted, PEmax% predicted, and PImax% predicted **(**Fig. 5**)**. The number of chewing cycles correlated negatively with PImax% predicted (*r* = -0.4; *p* = 0.0008; power = 0.7) and PEmax% predicted (*r* = -0.3; *p* = 0.02; power = 0.6). Lung volume measured through FVC% predicted correlated negatively with an increased solid swallowing time (*r* = -0.3; *p* = 0.02; power = 0.6), suggesting the need for more time to perform solid swallowing as pulmonary function worsens.

**Fig. 6:**
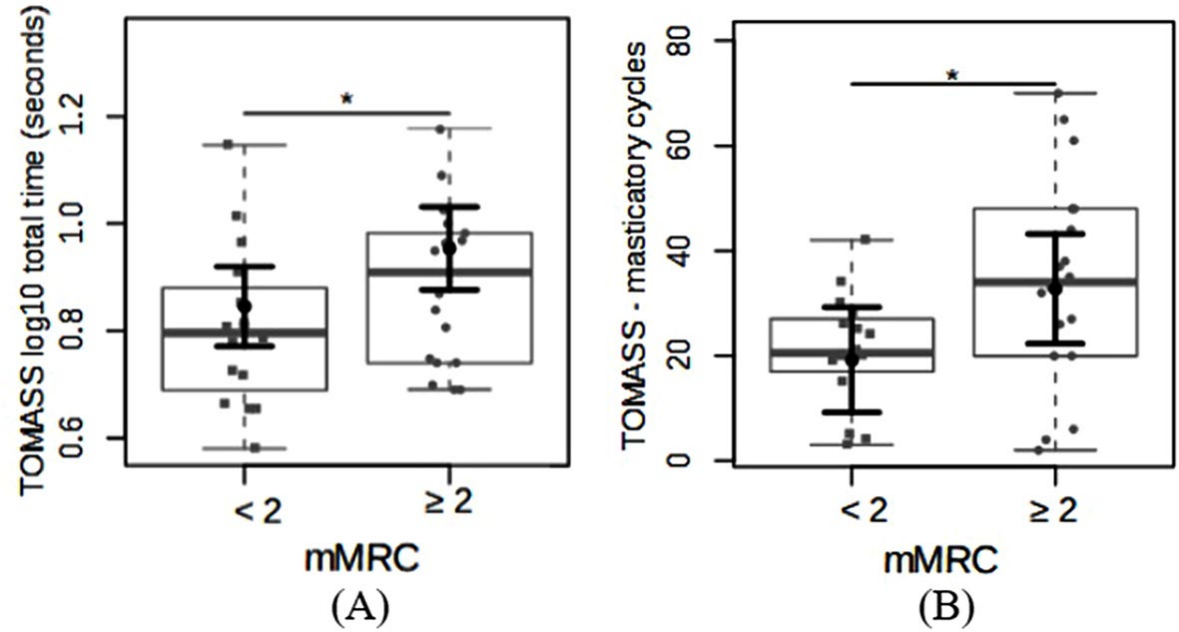
Differences for (**A**) the Test of Mastication and Swallowing Solids (TOMASS) total time (log 10) and (**B**) the TOMASS masticatory cycles between patients with interstitial pulmonary fibrosis and with or without dyspnea. The data are plotted in box plots and strip charts in gray. In black, the central circle represents the estimated mean marginal effect expected for each group estimated from linear fixed-effects models. The confounding variables were age, sex, body mass index, and the use of specific medications. The black horizontal bars represent the 95% confidence intervals of the expected mean marginal effects per group. Abbreviation: mMRC = Modified Medical Research Council

Swallowing solids was also impacted by dyspnea. Individuals without dyspnea complaints (mMRC < 2) had a longer time (log_10_) per swallow (log_10_ fold-change 0.84 ± 0.03 vs. 0.95 ± 0.03) and fewer chewing cycles (19.21 ± 4.88 vs. 32.73 ± 5.09) than individuals with dyspnea (mMRC ≥ 2). The mean difference in time (log_10_) was 0.1 ± 0.04 s (power = 0.6), and the mean difference in chewing cycles was 13.51 ± 6.39 (*p* < 0.05; power = 0.5; Fig. 6A, B).

The mean tongue pressure was 55.04 ± 16.11 kPa. We found no association between tongue pressure and BMI or liquid swallowing. However, individuals who had fewer swallows per bite than the median also exhibited statistically lower tongue pressure. In other words, those with a number of swallows per bite below the median demonstrated reduced tongue pressure. (53.26 ± 3.45 vs. 68.01 ± 6.21 kPa; mean difference 14.75 ± 6.33 kPa; *p* = 0.02)

## Discussion

The aim of this study was to investigate changes in swallowing of patients with IPF and their possible associations with the severity of this disease. We found that pulmonary function was related to swallowing, indicating that deteriorating lung function leads to adaptations in swallowing mechanics. In our sample, 5 individuals exhibited a risk of dysphagia based on the EAT-10. We attribute the limited representation of individuals at risk of dysphagia in our sample to the fact that few individuals exhibited an altered FVC.

We observed a weak but statistically significant correlation between alterations in liquid swallowing and pulmonary function (FEV1/FVC). Moreover, there was a negative correlation between masticatory cycles and respiratory muscle strength, as well as between lung volume (FVC) and the total swallowing time for solids. Lindh et al. [29] also described associations between alterations in lung function and swallowing in patients with pulmonary disease. Prolonged times for swallowing both solids and liquids indicate that our sample had a reduced swallowing efficiency and impaired masticatory ability. We believe that the findings related to solid swallowing in our study are attributed to individuals experiencing dyspnea before the evaluation. With the physiological increase in the respiratory frequency during chewing [30], they may have taken more pauses, requiring additional masticatory cycles for efficient chewing. Consequently, this led to an increase in the overall assessed time, as observed by Lindh et al. [31], who found an association between dyspnea and dysphagia.

Regarding the use of predicted FVC, predicted PImax, and FEV1/FVC as markers of IPF severity, our study revealed associations between worsening FVC and PImax and the presence of feeding adaptations. Moreover, an increase in FEV1/FVC, which is also associated with disease progression due to increased pulmonary elastic force resulting from fibrosis progression, correlated with a reduction in the liquid swallowing speed. This suggests that individuals with more severe IPF may experience altered swallowing mechanics. Notably, we observed these swallowing alterations even in individuals with mild-to-moderate disease, indicating that they may during the early stages of the disease.

In the literature, the swallowing speed is widely regarded as a reliable predictor of thin liquid tolerance across different patient populations [32, 33]. Consistent with previous studies by Nathadwarawala et al. [20] and Epiu et al. [34] in individuals with COPD, we found a reduced water swallowing speed in many of the evaluated individuals. This decrease in the swallowing speed is commonly observed in individuals with swallowing difficulties; it serves as a compensatory mechanism to minimize the risk of aspiration. Consequently, these individuals tend to reduce the bolus size, which in turn decreases the swallowing speed [20].

In addition to an increase in swallowing duration, there was a decrease in volume per swallow, indicating compensatory mechanisms or adaptations to dysphagia [35]. In our study, individuals classified as being at risk for dysphagia based on the EAT-10 exhibited a longer total swallowing time and a longer time per liquid swallow. Additionally, we observed longer swallowing durations among individuals who made feeding adaptations, suggesting the presence of ongoing modifications in swallowing patterns.

It is important to consider that factors such as sex and age can influence swallowing speed. To account for these variables, we utilized values standardized by Sarve et al. [22], who considered age and sex when establishing reference values. Regarding solid food swallowing, women showed longer durations in the TOMASS as well as more bites. This increased total swallowing time may be attributed to an increase in masticatory cycles resulting from weakened oropharyngeal musculature or reduced masticatory efficiency. While our study did not measure the strength of the masticatory muscles, we did assess tongue pressure and observed reduced pressure in the group with fewer swallows/bite. We hypothesize that this is due to individuals experiencing fatigue more rapidly, thereby reducing the number of masticatory cycles, or to their efficient chewing requiring fewer masticatory cycles. However, additional studies are needed to evaluate tongue resistance in individuals with IPF and to perform imaging examinations such as videofluoroscopy or videoendoscopy to analyze potential pharyngeal residues.

During chewing, healthy individuals experience alterations in the respiratory rhythm [4, 36]. The duration of the respiratory cycle, as well as the expiratory and inspiratory times, decreases significantly [30, 36], resulting in a 20% increase in respiratory frequency [4]. In our study, swallowing solids was associated with the number of masticatory cycles and the total swallowing time in relation to dyspnea. The individuals were already experiencing dyspnea prior to the evaluation, and the physiological increase in the respiratory frequency during chewing likely led to more pauses, requiring additional masticatory cycles to ensure efficient chewing and, consequently, increasing the total swallowing time.

Patients with chronic lung diseases often exhibit a clinical course characterized by progressive weight loss and muscle mass reduction [10, 37] due to increased energy expenditure associated with respiratory impairment, which can lead to malnutrition [10]. The deterioration in the nutritional status caused by IPF can result in a reduction in muscle mass, impacting swallowing and necessitating feeding adaptations. This was evident in our study, as lower scores on the nutritional questionnaire were significantly associated with the use of oxygen therapy and the implementation of feeding adaptations. Weight loss is a prognostic indicator of a poor outcome in patients with IPF [38]. Because our sample predominantly consisted of individuals with mild-to-moderate disease and a limited number of individuals with severe disease [28], all individuals presented a BMI > 18 kg/m^2^. Nevertheless, we observed a weak but statistically significant correlation between BMI and the volume/swallow ratio assessed in the TWST.

We acknowledge that this study has certain limitations, such as being conducted at a single center and including mostly individuals with mild-to-moderate IPF (due to the random inclusion of participants). Our protocol did not include imaging examinations for swallowing analysis (videofluoroscopy or videoendoscopy), which could have provided a better understanding of the observed adaptations. Nevertheless, we identified relationships between disease severity, swallowing alterations, and the nutritional status. Some of these relationships may be influenced by age, and future studies with a control group should be considered.

## Conclusions

At least in a subgroup of patients, the severity of IPF seems to be related to a poor nutritional status, the need for adaptations in eating, and swallowing of liquids. Patients with mild-to-moderate IPF already have a risk of dysphagia.

## Data Availability

The data that support the findings of this study are not openly available due to reasons of sensitivity and are available from the corresponding author upon reasonable request. Data are located in controlled access data storage at University of the State of Rio de Janeiro.
